# Outpatient management of children with chest indrawing pneumonia in primary healthcare settings in Punjab, Pakistan: a prospective cohort study

**DOI:** 10.7189/jogh.16.04139

**Published:** 2026-06-12

**Authors:** Zamir Hussain Suhag, Noorulain Altaf Khuwaja, Ashlesha Pal, Muhammad Naeem, Asad Raza Naqvi, Shamim Ahmad Qazi, Yasir Bin Nisar

**Affiliations:** 1 Trust for Vaccines & Immunization, Research Department, Karachi, Pakistan; 2 Department of Pathology and Laboratory Medicine, The Aga Khan University, Karachi, Pakistan; 3 Independent Consultant, Geneva, Switzerland; 4 Department of Sexual, Reproductive, Maternal, Child, Adolescent, and Ageing Health, World Health Organization, Geneva, Switzerland

## Abstract

**Background:**

Pneumonia remains a leading cause of mortality among children under five years of age in Pakistan. The government of Pakistan revised the national Integrated Management of Childhood Illness (IMCI) protocol in 2019, recommending outpatient treatment of pneumonia, characterised by fast breathing and/or chest indrawing, with oral amoxicillin in children aged 2–59 months. We aimed to evaluate parents/caregivers-reported outcomes in children presenting with chest indrawing pneumonia at primary healthcare (PHC) facilities of Lahore, Punjab, Pakistan.

**Methods:**

We conducted a prospective observational cohort study from 3 January 2024 to 28 February 2025 at five PHC facilities in the peri-urban areas of Lahore. We enrolled children aged 2–59 months with chest indrawing pneumonia and followed them up on day 15 post-enrolment. The primary outcome was the case-fatality rate (CFR), and the secondary outcomes included antibiotic use, treatment adherence, and hospitalisation. Two physicians conducted verbal autopsies independently for children who died during the period of follow-up to determine the cause of death. We calculated CFRs with 95% confidence intervals (95% CI).

**Results:**

We enrolled 360 children aged 2–59 months, of whom 356 (98.8%) completed the 15-day follow-up. The CFR for chest indrawing pneumonia was 0.6% (n/N = 2/356; 95% CI = 0.1–2.2). At follow-up, parents/caregivers reported that 336/356 (94.4%) children were cured, while 18/356 (5.0%) showed no improvement or had worsened health. Healthcare workers prescribed oral amoxicillin to 192/356 (53.9%) children, of whom 160/192 (83.3%) adhered to the specified duration of four or more days. On day 15 of follow-up, 26/356 (7.3%) parents/caregivers reported that they had switched to other antibiotics during treatment. Among these, 7/26 (26.9%) were hospitalised and received injectable antibiotics, 5/26 (19.2%) were managed as outpatients with injectable antibiotics, and 14/26 (53.8%) were treated with different oral antibiotics on an outpatient basis.

**Conclusions:**

The low CFR and high cure rate among children with chest indrawing pneumonia treated on an outpatient basis with oral antibiotics at the PHC settings in Lahore support the IMCI protocol. Management of chest indrawing pneumonia on an outpatient basis at the primary care level is feasible, with the potential to reduce unnecessary hospital referrals, reduce antimicrobial resistance, lower healthcare costs, and decrease mortality, particularly in resource-constrained settings.

**Registration:**

ISRCTN: ISRCTN12687253.

Global mortality in children under-five years of age has declined substantially over the past three decades, from 90 deaths per 1000 live births in 1990 to 37 per 1000 live births in 2023, yet progress remains insufficient to meet the 2030 Sustainable Development Goals target of ending preventable child deaths [1–6]. Pneumonia continues to be a major contributor to this phenomenon, causing more than 700 000 deaths annually and accounting for 14% of global mortality for children under five years of age [7]. Pakistan remains among the highest-burden countries, with an under-five mortality rate of 61 per 1000 live births, including seven pneumonia-related deaths per 1000 live births [8].

To improve access to timely and effective treatment, the 2012 guidelines published by the World Health Organization (WHO) simplified pneumonia classification and recommended outpatient management with oral amoxicillin for children aged 2–59 months with chest indrawing, but no danger signs [9–11]. Pakistan incorporated these recommendations into its national Integrated Management of Childhood Illness (IMCI) protocol in 2019 [12]. However, uptake remains variable; a WHO policy survey reported that fewer than one-third of countries had fully updated national protocols to support outpatient management with oral amoxicillin [13]. Concerns persist regarding whether outpatient treatment of chest indrawing pneumonia is safe in routine programmatic settings, especially in contexts with high prevalence of malnutrition, anaemia, or delayed care seeking [14]. Evidence from real-world implementation is, therefore, essential for understanding how IMCI recommendations are practised and whether outcomes remain favourable under routine PHC conditions [15]. In this context, we aimed to evaluate the management and clinical outcomes of children aged 2–59 months with chest indrawing pneumonia presenting to public-sector primary healthcare facilities in peri-urban Lahore, Pakistan. By describing treatment patterns, adherence, hospitalisation rates, and parents/caregivers-reported outcomes, we provide programmatically relevant evidence to guide improved pneumonia care delivery in similar low- and middle-income countries (LMICs).

## METHODS

This research was part of a multi-country study evaluating current practices in the outpatient management of chest indrawing pneumonia in low- and middle-income countries (LMICs).

### Study design and setting

**Figure 1:**
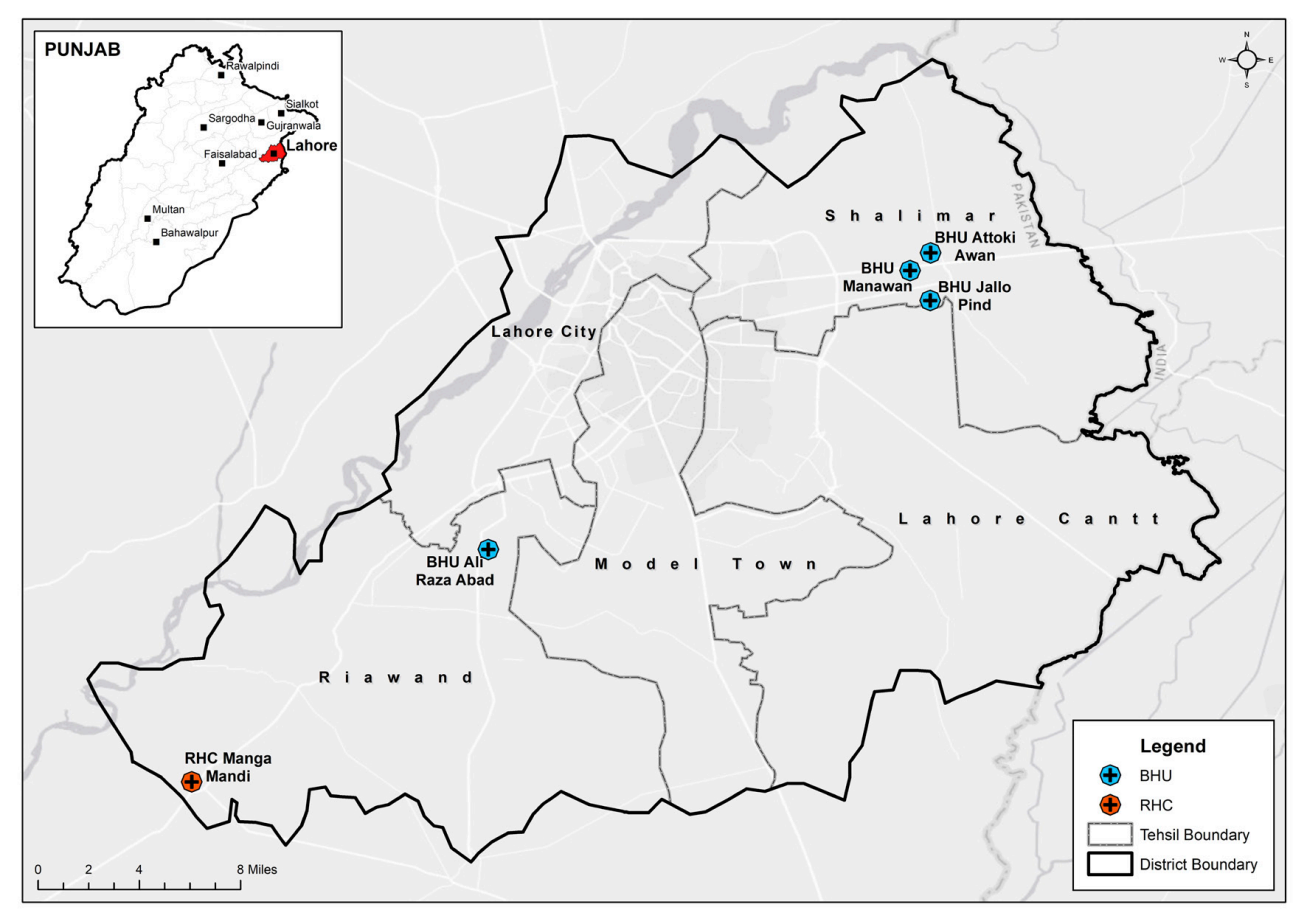
The map shows selected primary healthcare facilities in the Lahore district.

We conducted a prospective, observational cohort study in a programmatic setting at selected PHC facilities across peri-urban areas of Lahore District, Punjab, Pakistan. We selected facilities with high outpatient volume for children under five years of age. The study sites were the Rural Health Centre (RHC) Manga Mandi and Basic Health Units (BHU) Ali Raza Abad, BHU Attoki Awan, and BHU Jallo Pind. We added BHU Manawan in mid-October 2024 to meet the target sample size (**Figure 1**).

### Participants

We screened children aged 2–59 months who presented at a selected PHC facility with a history of cough and/or difficult breathing for eligibility. We included children with lower chest indrawing pneumonia, without danger signs and living within the catchment areas of the study sites to ensure the feasibility of follow-up. We excluded children with any general danger signs (including convulsions, inability to drink/feed, vomiting everything, lethargy, or unconsciousness), oxygen saturation (SpO_2_) < 90%, stridor when calm, or children whose parents did not consent to participate or were enrolled in another clinical study.

### Study procedures

#### Preparatory phase

We developed standardised questionnaires in the English language and translated them into the local language, Urdu. We implemented a digital system utilising an electronic data collection application to manage data in real-time.

IMCI master trainers conducted a three-day refresher training on the pneumonia component of the IMCI chart booklet for facility-based healthcare providers (HCPs) to ensure adherence to the IMCI protocol. The training covered counting respiratory rate, observing chest indrawing, general danger signs, and assessing SpO_2_ using Masimo Rad 5v pulse oximeters (Masimo Corporation, Irvine, CA, USA). Data collectors received three days of training on using study tools, electronic handheld devices, and conducting anthropometric assessments. Subsequently, we conducted a pilot study from 4 December 2023 to 2 January 2024, by screening more than 200 children presenting with cough and/or difficult breathing to validate study tools, workflow, the digital system for data management, and study feasibility.

#### Selection of primary healthcare facilities

We performed a health facility assessment at nine PHC facilities, using the Service Provision Assessment tool [16] and the Service Availability and Readiness Assessment tool [17] to assess service availability and readiness, medicines and commodities availability. Moreover, we evaluated the availability of human resources, catchment population, and immunisation services at these facilities. With consensus, we selected five PHC facilities for the study: RHC Manga Mandi, BHU Ali Raza Abad, BHU Attoki Awan, BHU Jallo Pind and BHU Manawan.

#### Screening and enrolment

IMCI-trained HCPs at the selected PHC facilities screened all children aged 2–59 months presenting with cough and/or difficult breathing. The clinical assessment included respiratory rate counting per minute using a respiratory rate timer and measurements of SpO_2_ using pulse oximeters with infant probes, as well as axillary temperature measurements using digital thermometers. The HCPs classified children into three mutually exclusive categories according to IMCI chart booklet: no pneumonia (cough/cold only), pneumonia (fast breathing pneumonia defined as ≥50 breaths per minute in 2–11-month-old infants and ≥40 breaths per minute in 12–59-month-old children) and/or chest indrawing pneumonia and severe pneumonia/severe disease defined as presence of any general danger sign (inability to drink or breastfeed, vomiting everything, convulsions, lethargy or unconsciousness), stridor in a calm child or hypoxaemia (SpO_2_<90%). Following the HCPs’ assessment, children classified as having chest indrawing pneumonia were enrolled by trained study staff after written informed consent was obtained from their caregivers. During enrolment, they collected detailed contact information, including the home address, parents/caregivers phone numbers, and landmark descriptions. They recorded anthropometric data at the time of screening, such as weight, height or length, and mid-upper arm circumference (MUAC). With the parent’s/caregiver's permission, we also recorded a video clip excluding the child’s face to document clinical signs such as chest indrawing.

The project manager conducted periodic validation of study data at both the PHC facility and household levels. At PHCs, we validated 2.4% (n/N = 165/6700) of children to assess HCPs' adherence to the IMCI chart booklet for assessment, classification, and management during monitoring and supervision. In the event of any discrepancy, it was addressed by providing refresher training. At the household level, 45.5% (n/N = 162/356) of the children who were followed up in the study were validated to verify PHC facility visits, treatment prescribed by HCP, treatment received, and the data collector's household visit.

#### Follow-up and outcome assessment

Data collectors conducted a systematic follow-up of all enrolled children on day 15 after enrolment, allowing a permissible window of ±2 days. We contacted the parents/caregivers 1–2 days before the follow-up visit by telephone to confirm their home addresses and availability. When parents/caregivers were challenging to reach, or the provided addresses were unclear, local community healthcare personnel – including lady health workers, lady health visitors, and vaccinators affiliated with the Expanded Programme on Immunization (EPI) were engaged to assist in locating the correct households for follow-up. We collected follow-up data electronically using handheld devices. We obtained detailed information from parents/caregivers on the child’s status at day 15, including whether the child had recovered, remained the same, worsened, or died. The clinical outcome was entirely based on parent/caregiver information. Parents/caregivers were asked to report the child’s condition as “cured,” “the same,” or “worse”; these categories reflected their perceived resolution, lack of change, or deterioration in the child’s health compared with the time of enrolment.

We collected additional data, including the treatment received, names of antibiotics, route of administration, duration, frequency, and adherence to the prescribed treatment. We documented relevant details for hospitalised children and verified antibiotic use through parents/caregiver’s confirmation of the prescription provided by the HCP during the initial visit at the PHC facility. If a prescription was unavailable, data collectors asked to see medication bottles or leaflets to recognise the prescribed and used medications. When neither was available, a pictorial chart of commonly used antibiotics for pneumonia was shown to parents/caregivers to identify them.

Parental education was documented by categorising whether the mother, father, or both were illiterate, able to provide a signature only, or had completed primary (up to 5 years of studies), secondary (between 6 and 10 years of studies), higher secondary education (between 11 and 12 years of studies) or graduation and above. The child's immunisation status was verified through vaccination cards when available; otherwise, caregiver recall was used. We asked the respondents specific questions regarding the timing of age-appropriate vaccinations according to the National EPI schedule, the route and site of administration, and whether they observed a scar mark on the child's body in case of Bacillus Calmette–Guérin vaccination. In cases of child’s death, a verbal autopsy was conducted after 4–6 weeks, using the WHO 2016 verbal autopsy questionnaire [18] for children aged 4 weeks to 14 years, alongside detailed parent/caregiver narratives to comprehensively document the events leading up to the child's death. Two qualified physicians independently determined the probable cause of death; no automated algorithms were applied due to the small number of deaths.

### Sample size

We calculated the sample size for the descriptive analysis to estimate the case fatality ratio (CFR) among children aged 2–59 months with chest indrawing pneumonia at each study site. The calculation was based on the standard single-proportion formula:



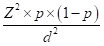



Where *p* is the expected CFR (5%, based on primary care data from Malawi), *Z* is 1.96 (corresponding to a 95% confidence level (95% CI)), and *d* is the desired precision (margin of error), applied as 3.0%. These parameters yielded a required sample size of 292 children.

To account for an anticipated 5% loss to follow-up, the sample size was adjusted as follows:



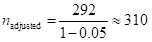



Therefore, our total sample size was 310 children aged 2–59 months with chest indrawing pneumonia. This target applied to our study site as a whole, rather than to each health facility. Including a design effect of 1.15, we arrived at a sample size of 356, which we rounded off to 360 [19].

### Outcomes

We collected data on primary and secondary outcomes on day 15 through parent/caregiver reports during the follow-up visit. The primary outcome was to determine the CFR among children aged 2–59 months who presented with chest indrawing pneumonia at the selected PHC facility. Secondary outcomes included assessing the type and duration of antibiotic treatment received, as well as adherence to the prescribed regimen. In addition, we aimed to determine the proportion of children who were hospitalised for chest indrawing pneumonia and to document the treatment they received.

### Data analysis

We examined the data for completeness before analysis. We described the baseline characteristics of children aged 2–59 months with chest indrawing pneumonia using frequencies and percentages for categorical variables. For continuous variables, we reported means and standard deviations (SD), as well as medians and interquartile ranges (IQR). The primary outcome, CFR, was determined by dividing the number of deaths recorded on day 15 post-enrolment by the total number of enrolled children who completed follow-up, with a 95% CI. To evaluate the potential impact of missing outcome data on the CFR, we conducted a simple sensitivity analysis for the four children who were lost to follow-up. The primary analysis followed a complete-case approach, excluding these four children from the denominator. For the sensitivity analysis, we recalculated the CFR under two extreme assumptions to assess the possible range of mortality estimates: a worst-case scenario in which all four missing children were assumed to have died; and a best-case scenario in which all four were assumed to have recovered.

We defined adherence as the administration of oral amoxicillin for 4–5 days or more. We calculated anthropometric indices as z-scores based on the 2006 WHO growth reference standards [20]. We considered children fully immunised if they had received all age-appropriate vaccinations according to Pakistan's National EPI schedule. Two physicians independently reviewed verbal autopsy data to assign the cause of death. If the two assessments disagreed, we consulted a third expert physician to resolve discrepancies and establish the most probable cause of death. We used Stata, version 17.0 (StataCorp LLC, College Station, TX, USA) for analysis.

## RESULTS

**Figure 2:**
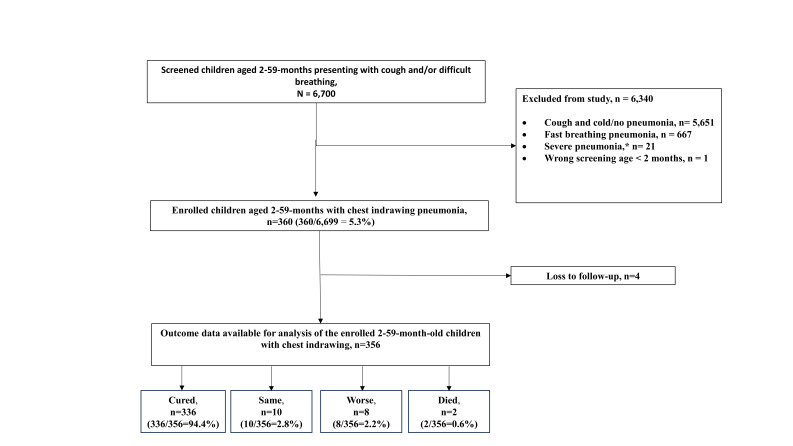
Flowchart of the study. *Severe pneumonia: presence of general danger sign (unable to eat or drink, vomits everything, convulsions or history of convulsions, lethargic or unconscious), stridor in a calm child; hypoxaemia (oxygen saturation using pulse oximetry SpO_2_ < 90%).

**Figure 3:**
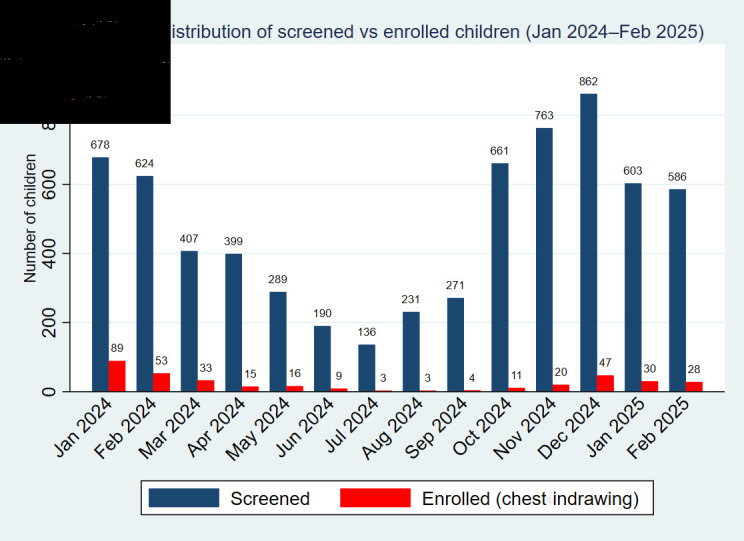
Monthly distribution of all children screened for cough, cold, and/or difficult breathing and the subset enrolled with chest indrawing pneumonia across five primary healthcare facilities (January 2024–February 2025).

A total of 6,700 children presenting with cough and/or difficult breathing were screened at the selected primary healthcare facilities between 3 January 2024 and 28 February 2025. One screened child was younger than two months and was therefore excluded as ineligible from the analysis. Of the remaining 6,699 eligible children aged 2–59 months, 360 children with chest indrawing pneumonia were enrolled. Follow-up data were available for 356 children, while four children were lost to follow-up because the study team was unable to contact their parents/caregivers or locate their households (**Figure 2**). During the study period, the monthly number of children screened and enrolled varied in line with the seasonal pattern of acute respiratory infections in Pakistan. ARI/pneumonia cases were higher during the winter months, particularly from November to February, and declined thereafter, with comparatively fewer cases observed during the summer months. The month-wise distribution of screened and enrolled children reflects these seasonal fluctuations (**Figure 3**).

### Baseline demographic and clinical characteristics

The highest number of participants was enrolled at RHC Manga Mandi (n = 132, 36.7%), followed by BHU Ali Raza Abad (n = 90, 25.0%). Among the 360 enrolled children, 193 (53.6%) were aged 2–5 months, with a mean age of 9.1 months (SD = 9.5), and 202 (56.1%) were male. The mean respiratory rate was 59 breaths/min (SD = 7.9) for children aged 2–11 months. The mean SpO_2_ was 96.0% (SD = 2.2%). Most children were malnourished; 81 (22.5%) had a weight-for-age z-score (WAZ)<–3, 66 (18.3%) had a height-for-age z-score (stunted)<–3, and 13.9% had a weight-for-length z-score (severe acute malnutrition) <–3. MUAC was measured in 167 children aged 6–59 months, and only 9 (5.3%) had a MUAC of ≤11.5 cm. Both parents were educated in 189 (52.5%) children. A total of 210 (58.3%) were fully immunised (**Table 1**). The study was not powered to detect differences across nutritional or immunisation strata, nor to assess statistical associations between these characteristics and clinical outcomes. However, we have included stratified descriptive summaries to enhance transparency (Table S1 in the **Online Supplementary Document**).

**Table 1:** Baseline demographic and clinical characteristics of children aged 2–59 months with chest indrawing pneumonia (n = 360)*

	n (%)
**Name of the participating health facility**	
RHC Manga Mandi	132 (36.7)
BHU Ali Raza Abad	90 (25.0)
BHU Jallo Pind	55 (15.3)
BHU Attoki Awan	42 (11.7)
BHU Manawan	41 (11.4)
**Age in months**	
x̄ (SD)	9.1 (9.5)
MD (IQR)	5.4 (3.8–9.6)
**Age category in months**	
2–5	193 (53.6)
6–11	94 (26.1)
12–59	73 (20.3)
**Sex of the child**	
Male	202 (56.1)
Female	158 (43.9)
**Axillary temperature in °C**	
x̄ (SD)	36.9 (0.6)
MD (IQR)	36.8 (36.7, 37.2)
**Temperature in °C**	
<38.0	340 (94.4)
≥38.0	20 (5.6)
**Respiratory rate/min (age 2–11 months)**	
x̄ (SD)	58.6 (7.9)
MD (IQR)	57.0 (53.0, 63.0)
**Respiratory rate/min (age 12–59 months)**	
x̄ (SD)	52.3 (9.6)
MD (IQR)	52.0 (46.0, 57.0)
**Oxygen saturation (SpO2) %**	
x̄ (SD)	96.0 (2.2)
MD (IQR)	97.0 (94.0-98.0)
**Oxygen saturation (SpO2) in % (age 2–59 months)**	
90–93	61 (16.9)
94–100	299 (83.1)
**Oxygen saturation (SpO2) in % (age 2**–**5 months), n = 193**	
90–93	34 (17.6)
94–100	159 (82.4)
**Oxygen saturation (SpO2) in % (age 6**–**11 months), n = 94**	
90–93	11 (11.7)
94–100	83 (88.3)
**Oxygen saturation (SpO2) in % (age 12**–**59 months), n = 73**	
90–93	16 (21.9)
94–100	57 (78.1)
**Classification of enrolled children**	
Chest indrawing pneumonia	9 (2.5)
Chest indrawing pneumonia along with fast breathing	351 (97.5)
**Anthropometry**	
WAZ	
*<−3*	81 (22.5)
*−3≤WAZ<−2*	62 (17.2)
*≥−2*	214 (59.4)
*Flag (WAZ<−6 or WAZ>5)*	3 (0.8)
WLZ	
*<−3*	50 (13.9)
*−3≤WLZ<−2*	44 (12.2)
*≥−2*	260 (72.2)
*Flag (WLZ<−5 or WLZ>5)*	6 (1.7)
HAZ	
*<−3*	66 (18.3)
*−3≤HAZ<−2*	54 (15.0)
*≥−2*	230 (63.9)
*Flag (HAZ<−6 or HAZ>6)*	10 (2.8)
MUAC in cm (n = 167)†	
*≥12.5*	154 (92.2)
*11.5–<12.5*	4 (2.3)
*≤11.5*	9 (5.3)
Immunisation status	
*Fully immunised‡*	210 (58.3)
*Partially immunised*	136 (37.8)
*Not immunised*	10 (2.8)
*Missing§*	4 (1.1)
Adult primary respondent	
*Mother*	312 (86.7)
*Father*	34 (9.4)
*Grandmother*	7 (1.9)
Other**	3 (0.8)
*Missing§*	4 (1.1)
Parental education¶	
*Both uneducated*	73 (20.3)
*Both educated*	189 (52.5)
*Only father educated*	44 (12.2)
*Only mother educated*	50 (13.9)
*Missing§*	4 (1.1)

BHU – basic health unit, RHC – rural health centre, IQR – interquartile range, MD – median, SD – standard deviation, WAZ – weight-for-age z-score, WLZ – weight-for-length/height z-score, HAZ – height-for-age z-score, MUAC – mid-upper arm circumference, x̄ – mean

*Presented as n (%) unless specified otherwise.

**†**We measured MUAC only in children aged 6 months or older.

**‡**Fully immunised child is defined as a child who has received all age-appropriate vaccines: Bacillus Calmette–Guérin dose at birth, four doses of polio, one dose of inactivated poliovirus vaccine, trivalent doses of Penta, three doses of pneumococcal conjugate vaccine, two doses of Rota, and one dose of measles.

**§**Children lost to follow-up.

¶Parental education was classified as educated if they had primary education or higher, and uneducated if illiterate or able to sign only. Categories reflect combined parental education status.

**Two aunts and one sister.

### Primary outcome

Of the 360 enrolled children, 356 (98.8%) completed follow-up on day 15. According to parents’/caregivers’ reports, 336 (94.4%) recovered and only two (CFR = 0.6%; 95% CI = 0.1–2.2) infants younger than six months died (**Table 2**, **Box 1**). Based on the sensitivity analysis, the worst-case scenario, assuming all four children lost to follow-up had died, the CFR (95% CI) increased to 1.7% (n/N = 6/360; 95% CI = 0.6–3.6), whereas under the best-case scenario, assuming all four had recovered, CFR remained 0.6% (n/N = 2/360; 95% CI = 0.1–2.0).

**Table 2:** Treatment outcomes reported by parents/caregivers in enrolled followed-up children (n = 356)

Outcome	Total		2–5 months (n = 190)		6–11 months (n = 94)		12–59 months (n = 72)	
**n (%)**	**(95% CI)**	**n (%)**	**(95% CI)**	**n (%)**	**(95% CI)**	**n (%)**	**(95% CI)**
Cured	336 (94.4)	(91.4–96.3)	178 (93.7)	(89.2–96.4)	89 (94.7)	(87.8–97.8)	69 (95.8)	(87.6–98.7)
Same	10 (2.8)	(1.5–5.1)	5 (2.6)	(1.1–6.2)	4 (4.2)	(1.6–10.9)	1 (1.4)	(0.2–9.5)
Worse	8 (2.2)	(1.1–4.4)	5 (2.6)	(1.1–6.2)	1 (1.1)	(0.1–7.3)	2 (2.8)	(0.6–10.7)
Died	2 (0.6)	(0.1–2.2)	2 (1.1)	(0.2–4.1)				

CI – confidence interval

Box 1 **Verbal autopsy details of deaths**

**Case 1**

A three-month and 14-day-old male infant presented to a selected PHC facility with a one-day history of fever, cough, and/or difficulty in breathing. He was screened and classified with chest indrawing pneumonia and managed as an outpatient with a five-day course of oral amoxicillin-clavulanic acid. At enrolment, the child’s respiratory rate was 60 breaths per minute, oxygen saturation (SpO_2_) was 92%, temperature was 37.8°C, weight was 4.5 kg, and length was 55 cm. His weight-for-height z-score was −0.12, and he had received all age-appropriate vaccinations.

The parents reported no improvement in the child’s condition. They subsequently sought care from a private clinic, where he received unspecified outpatient treatment. According to the parents, his condition continued to worsen, prompting a second visit to the same private clinic. Upon reassessment, the HCP referred the child to the same PHC facility where he was initially enrolled.

On day 14 of illness, the child re-visited the PHC facility and was immediately referred to a tertiary care hospital. The parents reported that his initial symptoms – fever, cough, and respiratory difficulty – had persisted throughout the illness. Upon arrival at the tertiary care hospital, the child’s condition further deteriorated. He was admitted, and treatment was initiated, including injectable medications and a blood transfusion. However, the parents could not recall specific details of the investigations or treatment administered during the hospital stay.

The mother reported that a nasogastric (NG) tube was inserted and that blood was observed in the NG tube. She also noted the onset of abdominal distension and an absence of urine output before the child’s death. The child was later transferred to the intensive care unit, where he was placed on mechanical ventilation. Despite these efforts, he did not survive and passed away on the 15th day of illness. Based on the verbal autopsy independently reviewed by two physicians, the most likely cause of death was septicaemia.

**Case 2**

A three-month-old male infant presented to a selected PHC facility with an eight-day history of cough and difficulty in breathing, along with a three-day history of high-grade fever. He was screened and classified as chest indrawing pneumonia according to IMCI protocol and was prescribed a five-day course of oral cefixime for outpatient management. At the time of enrolment, his respiratory rate was 74 breaths per minute, SpO_2_ was 93%, and body temperature was 37.0°C. His weight was 3.6 kg, length 56 cm, and weight-for-height z-score was −3.58. The child was partially immunised for his age.

According to the parents, there was no improvement in the child's condition following the prescribed treatment. The next day, they sought care at a private clinic, where some investigations were conducted, and oral medications (names unknown) were prescribed.

Despite the change in treatment, the child’s condition continued to deteriorate. Subsequently, he was taken to a private hospital (exact time unknown), where he was administered injectable medications and received a blood transfusion. The parents reported that the child showed slight improvement during the transfusion; however, shortly after its completion, his condition worsened. He began gasping, and his eyes rolled upward. Believing that the child had passed away, the parents alerted the HCP. Upon examination, the HCP confirmed that the child was still alive and advised immediate referral to a tertiary care hospital. The child was promptly transferred to the tertiary care hospital. However, upon arrival, the attending physician informed the parents that the child had died. Based on data collected through verbal autopsy and independently reviewed by two physicians, the most probable cause of death was determined to be severe pneumonia.

### Secondary outcomes

#### Antibiotic prescription

Among 356 followed-up children, all were prescribed at least one oral antibiotic at the time of enrolment. Oral amoxicillin was prescribed for 192/356 (53.9%) children across all PHC facilities. The prescription frequency was highest at BHU Manawan (n/N = 30/41, 73.2%), followed by BHU Jallo Pind (n/N = 38/54, 70.4%) and Ali Raza Abad (n/N = 58/89, 65.2%). The prescription rate for oral amoxicillin was lowest at RHC Manga Mandi (n/N = 42/131, 32.1%). Oral cefixime was prescribed in n/N = 58/356 (16.3%) and oral amoxicillin-clavulanic acid in n/N = 54/356 (15.2%) children, mainly at RHC Manga Mandi (Table S2 in the **Online Supplementary Document**).

#### Duration of antibiotic prescription and adherence patterns

The PHC facilities’ HCPs prescribed oral amoxicillin for 4–5 days in 166 out of 192 (86.5%) children. Parents/caregivers administered oral amoxicillin for 4–5 days in 125/166 (75.3%) children, while 35/166 (21.1%) children were given the antibiotic for more than 5 days. Children who received fewer than 4 days of amoxicillin were classified as non-adherent, in accordance with WHO-recommended treatment duration. Overall, 160/166 (96.3%) adhered to the prescribed course of oral amoxicillin (**Table 3**).

**Table 3:** Duration of antibiotic treatment prescribed by the healthcare provider at the primary healthcare facility and administered by parents/caregivers in successfully followed-up children (n = 356)*

		Name of antibiotics prescribed by HCP				
	**Total, n = 356**	**Oral amoxicillin, n = 192**	**Oral cefixime, n = 58**	**Oral amoxicillin-clavulanic acid, n = 54**	**Oral cotrimoxazole, n = 33**	**Other oral antibiotics, n = 19†**
**Treatment duration in days prescribed by HCP**						
1–3	67 (18.8)	26 (13.5)	14 (24.1)	5 (9.3)	11 (33.3)	11 (57.9)
4–5	289 (81.2)	166 (86.5)	44 (75.9)	49 (90.7)	22 (66.7)	8 (42.1)
**Treatment duration in days administered by parents/caregivers**						
1–3	79 (22.2)	32 (16.7)	14 (24.1)	9 (16.7)	11 (33.3)	13 (68.4)
4–5	209 (58.7)	125 (65.1)	31 (53.4)	28 (51.9)	20 (60.6)	5 (26.3)
>5	68 (19.1)	35 (18.2)	13 (22.4)	17 (31.5)	2 (6.1)	1 (5.3)

HCP – healthcare provider

*Presented as n (%).

†Other oral antibiotics include azithromycin (n = 15), doxycycline (n = 3), and cefadroxil (n = 1).

#### Hospitalisations

Among 356 children, 7 (2.0%; 95% CI = 0.8, 4.0) were hospitalised during the 15-day follow-up period. Parents/caregivers self-referred four of these seven admissions (57%), PHC providers initiated two, and a community health worker referred one. Three admissions occurred within the first three days post-enrolment, while four occurred later in the follow-up period (Table S3 in the **Online Supplementary Document**).

#### Treatment change during the follow-up period

According to parents/caregivers, 26/356 (7.3%) children reported a change in their antibiotic treatment during the 15-day follow-up period. Of these, 7 (26.9%) were hospitalised and received injectable antibiotics, 5 (19.2%) received injectable antibiotics on an outpatient basis, and 14 (53.8%) were switched to alternative oral antibiotics as an outpatient (Table S3 in the **Online Supplementary Document**). Among these 26 children, 14 were initially prescribed oral amoxicillin at enrolment, of whom 10 (71.4%) completed the full prescribed course. Among the 10 children who adhered to the initially prescribed course of oral amoxicillin, treatment changes occurred at different time points after enrolment. Three children (30.0%) had a change in treatment within up to three days of enrolment (including two early escalations and one switch to symptomatic cough treatment). Two children (20.0%) had a change between day three and day five, and another two children (20.0%) had initiated a new antibiotic treatment between day six and day nine. The remaining three children (30.0%) had initiated a new antibiotic treatment on day 10 or later (Table S3 in the **Online Supplementary Document**).

## DISCUSSION

This prospective observational cohort study showed a low CFR (0.6%) by day 15 post-enrolment among 2–59 months old children with chest indrawing pneumonia when managed at selected PHC facilities in Lahore, Pakistan. Parents/caregivers reported that nearly 95% of enrolled children were cured at the time of follow-up on day 15. The HCPs at the selected PHC facilities prescribed oral antibiotics to all enrolled children; over half (54%) were prescribed oral amoxicillin, which is recommended as the first-line treatment for chest indrawing pneumonia in children aged 2–59 months by both the WHO and Pakistan's national IMCI 2019 [12], while the rest were prescribed an alternative oral antibiotic.

Comparison of CFR across observational studies of chest indrawing pneumonia shows uniformly low mortality. In our study, the observed CFR closely aligns with the 0.4% reported in Thatta district, Sindh province, Pakistan, and 0.6% in the North Gondar zone, Amhara region, Ethiopia, and findings from methodologically comparable studies implemented in different geographic contexts [21,22]. Other observational studies that used oral antibiotics to treat chest indrawing pneumonia in children aged 2–59 months in Zambia, Papua New Guinea and a multi-country study from Bangladesh, Egypt, Ghana, and Vietnam reported no deaths. However, both the Papua New Guinea and the multi-country studies excluded children with severe malnutrition at enrolment [23–25]. In contrast, one community-based study from Kenya reported a CFR of 0.3% [26]. Three multi-site community-based randomised controlled trials, two conducted in Pakistan [27,28] and one in four countries named Bangladesh, Ethiopia, India, and Malawi [29], reported a CFR<0.5% where community-level health workers treated children aged 2–59 months with chest indrawing pneumonia with oral amoxicillin at home. Effective management of childhood pneumonia requires not only the appropriate antibiotic but also strong adherence to treatment protocols. We found that adherence to oral amoxicillin, the WHO and National IMCI-recommended first-line antibiotic, was over 95% when prescribed according to the IMCI protocol. This finding aligns with evidence from Kenya, showing similarly high adherence to amoxicillin in community settings [30]. However, contrasting patterns have been reported from Colombia and Vietnam, where irrational prescribing practices were observed [31,32]. These variations highlight the critical role of standardised case management and HCP training in ensuring appropriate antibiotic use. The WHO's Access, Watch, Reserve (AWaRe) classification framework [33] emphasises prioritising the use of ‘Access’ antibiotics, such as oral amoxicillin, for common infections, including childhood pneumonia, with a target of at least 60% of total antibiotic consumption globally should be from this group [33]. HCPs prescribed oral amoxicillin to 54% of children, compared to 62% in a similar study conducted in Thatta, Pakistan [21], and over 85% in Zambia [23]. In line with this, a study from the Amhara region of Ethiopia prescribed oral amoxicillin to all 345 enrolled children with chest indrawing pneumonia, highlighting full adherence to the IMCI protocol [22]. In contrast, in our study, the HCPs prescribed a considerable proportion of oral cefixime or amoxicillin-clavulanic acid, both categorised as ‘Watch’ antibiotics, which should be used more judiciously [33]. This deviation from the IMCI protocol may contribute to the growing threat of antimicrobial resistance (AMR), particularly in countries like Pakistan, where surveillance indicates a rising burden of multidrug-resistant infections [34]. Failure to prioritise ‘Access’ antibiotics not only undermines treatment effectiveness but also accelerates resistance by increasing selective pressure from broader-spectrum agents [35]. This concern is echoed in global paediatric prescribing data from 56 countries, where ‘Watch’ antibiotic use frequently exceeded ‘Access’ antibiotic use, particularly in LMICs [36]. While we did not measure antimicrobial resistance, strengthening outpatient management and promoting the use of first-line ‘Access’ antibiotics may indirectly contribute to reducing unnecessary broad-spectrum antibiotic use [33]. These findings emphasise the urgent need to reinforce training of HCPs at PHC facilities and strengthen the supply chain for the ‘Access’ group of antibiotics consistently, and the implementation of IMCI protocols to ensure evidence-based prescribing. Adopting these strategies will support both optimal clinical care and national AMR containment goals.

Outpatient management of chest indrawing pneumonia in children with oral amoxicillin offers cost-saving advantages, particularly in LMICs such as Pakistan. This strategy substantially reduces healthcare costs and alleviates burdens on overstretched health systems [37]. Sadruddin *et al.* reported an average household cost of USD 1.46 for pneumonia cases managed by community health workers compared to USD 7.60 for hospital referrals in Pakistan [38]. Similarly, societal costs were considerably lower for non-severe pneumonia treated on an outpatient basis (USD 22.6 per episode) compared to severe cases, which required inpatient care and cost USD 142.9 per episode, with medicines being the most considerable expense (40.5%), followed by meals (23.7%), hospitalisation (13.2%), and transport (12.2%) [39]. An economic review across 74 LMICs further demonstrated the cost-effectiveness of the updated 2012 WHO guidelines recommending oral amoxicillin outpatient management, estimating potential savings up to USD 1.16 billion [40]. These findings highlight the economic benefits of managing non-severe pneumonia in children as outpatients using the 2012 WHO-recommended guidelines [9].

According to parents/caregivers, 26/356 (7.3%) children reported switching to another treatment during the 15-day follow-up period. However, not all changes in antibiotic treatment occurring late in the follow-up period represent treatment failure. According to the WHO Pocket Book, children treated for pneumonia as outpatients should be reviewed after three days; lack of improvement by this point suggests possible treatment failure and warrants further evaluation [10]. Most hospital-based and specialty guidelines, including those from the Infectious Diseases Society of America and Paediatric Infectious Diseases Society, further note that children receiving appropriate antibiotics are expected to show clinical improvement within 48–72 hours [41]. We found that children who required a change in treatment more than a week after starting amoxicillin, particularly those switching after day 10 of enrolment, are thus unlikely to represent a failure of the recommended antibiotic, but more plausibly represent persistent symptoms, parent/caregiver concerns, or provider preference for antibiotic escalation. Additionally, a persistent cough after the resolution of pneumonia is well-documented and does not in itself indicate antibiotic failure. A cough lasting several weeks after apparent recovery from pneumonia may be due to post-infectious airway inflammation and does not, by itself, indicate failure of the initial antibiotic treatment [42]. Similarly, a change in antibiotic within 72 hours of enrolment likely represents premature escalation before adequate time had elapsed for the expected clinical response to amoxicillin. This distinction is crucial for clinicians to avoid unnecessary antibiotic escalation and to provide appropriate reassurance and guidance to parents/caregivers [41].

In our study, only seven children required hospitalisation, the majority of whom were admitted to public sector hospitals. In comparison, no hospitalisations were reported in the aforementioned study from Zambia [23], while the study from Ethiopia reported seven [22], and the study from Thatta, Pakistan, reported only two hospitalisations [21].

Hospitalisation was also uncommon (2%) in a study in peri-urban Karachi, where public hospitals were the predominant site of admission [43]. In our study, four of the seven hospitalised children (57.1%) were self-referred, suggesting that parents/caregivers were able to recognise clinical deterioration and initiate timely care-seeking without referral. The low proportion of hospitalisations reinforces the feasibility and safety of outpatient management of chest indrawing pneumonia at PHC facilities, using the IMCI protocol, but also the potential for appropriate caregiver response in the event of clinical worsening, thereby supporting the implementation of PHC-based pneumonia care models in similar resource-constrained settings.

The implementation of outpatient pneumonia management at PHC facilities offers several public health benefits and has several implications for health systems. First, outpatient care reduces the risk of hospital-acquired infections, which remain a significant cause of morbidity and mortality in hospitalised children, particularly in low-resource settings [44,45]. Second, this approach substantially lowers the economic burden on families and health systems. Studies from Pakistan have demonstrated that outpatient management significantly reduces household treatment costs compared to inpatient care, thereby alleviating financial stress and making healthcare more accessible [38,39]. Third, outpatient management reduces the burden on referral-level healthcare facilities, enabling these facilities to concentrate their resources on critically ill patients and complex medical conditions. Additionally, promoting rational use of first-line antibiotics such as amoxicillin at the PHC level has been shown to mitigate AMR by limiting unnecessary use of broad-spectrum antibiotics, preserving their effectiveness for severe infections [46]. Collectively, these findings underscore the clinical, economic, and public health rationale for adopting outpatient management protocols for childhood pneumonia, especially in resource-constrained settings such as Pakistan, where policymakers and health planners have a critical role in ensuring the consistent implementation of IMCI protocols through adequate training, reliable medicine supply, and effective supervision at PHC facilities. The implications of these findings apply specifically to non-hypoxaemic, carefully screened children with chest-indrawing pneumonia who are managed in settings where IMCI triage and referral practices are consistently followed. The low CFR observed in this study should therefore be interpreted within this context, and not generalised to children with danger signs or to cases of severe pneumonia.

### Strengths and limitations

This study has several strengths. The integration of this study into the PHC delivery system in the Lahore district enabled the real-time identification of pneumonia cases, reflecting the actual burden and care-seeking practices in the community. We successfully enrolled a substantial number of pneumonia cases, with no refusals to participate, which enhanced the reliability of our findings. The negligible loss to follow-up reduced the potential for attrition bias, thus ensuring the representativeness of the results. The use of a standardised case management IMCI protocol by the HCPs for assessing, classifying, and managing pneumonia was encouraging. These results could be reflective of similar socio-economic conditions and settings.

Our study also had a few limitations. First, we based the day-15 outcome solely on parent/caregiver-reported information, which introduces some degree of subjectivity and the potential for misclassification. However, parents/caregivers reported their child’s condition as ‘cured,’ ‘same,’ or ‘worse’ based on their current health status compared with the time of enrolment, without clinical or physiological verification. However, the 15-day follow-up period is generally sufficient for pneumonia to either resolve or progress to a severity that would prompt parents/caregivers to seek further care, thereby reducing the likelihood of major misclassification. The distribution of outcomes, with 94.4% participants reported as cured, 2.8% as unchanged, 2.2% as worsened, and 0.6% as deceased, is consistent with the pattern reported in previous trials of oral amoxicillin for chest-indrawing (or similar severity) pneumonia in Africa and Asia, which reported low rates of treatment failure and very low mortality among appropriately treated children, supporting the internal consistency and plausibility of caregiver–reported responses [29,47]. In addition, findings from a pragmatic trial in Kenya also support high recovery and very low mortality, although the study used the earlier WHO case definition of ‘severe pneumonia’, in which chest indrawing was included; its relevance is therefore not directly equivalent [48]. However, its findings still align with the expected favourable clinical trajectory of non-hypoxaemic chest-indrawing pneumonia managed with oral amoxicillin. To minimise reporting errors, data collectors used structured probing questions during follow-up. Nevertheless, some degree of misclassification or recall bias remains possible, mainly when the illness course or treatment details were inaccurately remembered. Second, the study period was marked by occasional stockouts of oral amoxicillin at the participating PHC facilities, which could have influenced prescribing behaviours at these sites. Such logistical constraints are typical in resource-constrained environments and may have contributed to the observed treatment patterns [49,50].

We performed the first evaluation of the outpatient management of chest indrawing pneumonia from Punjab province following the 2019 IMCI protocol revision in Pakistan. Conducted within routine public-sector primary care settings, it employed real-time electronic data capture and systematic verification, ensuring methodological rigour and data reliability. These features expand the national evidence base to a new provincial context, strengthening the understanding of IMCI implementation at the PHC level.

## CONCLUSIONS

Our findings support using the IMCI protocol to treat chest indrawing pneumonia in children aged 2–59 months with a five-day course of oral amoxicillin on an outpatient basis. Based on our results, we recommend the large-scale adoption and implementation of the IMCI protocol. This recommendation has the potential to increase access to treatment, improve treatment coverage, reduce referrals and pressure on overburdened referral health facilities, lower costs for the health system and families, and subsequently curtail childhood pneumonia-related deaths in resource-limited settings. IMCI-trained healthcare providers can safely manage childhood pneumonia without danger signs at the PHC level, while ensuring timely referral for severe pneumonia/disease, and with sustained availability of essential antibiotics. Further studies are required to examine the barriers and facilitators for the effective adoption of the IMCI protocol and to assess real-world feasibility and long-term sustainability.

## Additional material


Online Supplementary Document

## References

[1] Bhusal MK Khanal SP A systematic review of factors associated with under-five child mortality. BioMed Res Int. 2022;2022:1181409. 10.1155/2022/1181409 36518629 PMC9744612

[2] World Health Organization. Millennium Development Goals (MDGs). Geneva, Switzerland: World Health Organization; 2018. Available: https://www.who.int/news-room/fact-sheets/detail/millennium-development-goals-%28mdgs%29. Accessed: 12 June 2025.

[3] United Nations Inter-Agency Group for Child Mortality Estimation. Levels and trends in child mortality: Report 2015. New York, USA: United Nations Children’s Fund; 2015. Available: https://www.un.org/development/desa/pd/sites/www.un.org.development.desa.pd/files/un-igme-child-mortality-report-2015.pdf. Accessed: 12 June 2025.

[4] United Nations Inter-Agency Group for Child Mortality Estimation. Levels and trends in child mortality: Report 2024 – estimates developed by the United Nations Inter-Agency Group for Child Mortality Estimation. New York, USA: United Nations Children’s Fund; 2024. Available: https://childmortality.org/wp-content/uploads/2025/03/UNIGME-2024-Child-Mortality-Report.pdf. Accessed: 11 November 2025.

[5] World Health Organization. SDG target 3.2: by 2030, end preventable deaths of newborns and children under 5 years of age. 2025. Available: https://www.who.int/data/gho/data/themes/topics/indicator-groups/indicator-group-details/GHO/sdg-target-3.2-newborn-and-child-mortality. Accessed: 12 June 2025.

[6] Sharrow D Hug L You D Alkema L Black R Cousens S Global, regional, and national trends in under-5 mortality between 1990 and 2019 with scenario-based projections until 2030: a systematic analysis by the UN Inter-agency Group for Child Mortality Estimation. Lancet Glob Health. 2022;10:e195–206. 10.1016/S2214-109X(21)00515-5 35063111 PMC8789561

[7] The United Nations International Children’s Emergency FundPneumonia. 2024. Available: https://data.unicef.org/topic/child-health/pneumonia/. Accessed: 5 May 2025.

[8] The United Nations International Children’s Emergency Fund. UNICEF Data: Pakistan. 2019. Available: https://data.unicef.org/country/pak/. Accessed: 5 May 2025.

[9] World Health Organization. Recommendations for management of common childhood conditions: evidence for technical update of pocket book recommendations: newborn conditions, dysentery, pneumonia, oxygen use and delivery, common causes of fever, severe acute malnutrition and supportive care. Geneva, Switzerland: World Health Organization; 2012. Available: https://iris.who.int/server/api/core/bitstreams/b9f75861-794a-46f2-bf57-e6022a64426f/content. Accessed: 5 May 2025. 23720866

[10] World Health Organization. Pocket book of hospital care for children: guidelines for the management of common childhood illnesses. 2nd ed. Geneva, Switzerland: World Health Organization; 2013. Available: https://www.who.int/publications/i/item/978-92-4-154837-3. Accessed: 5 May 2025. 24006557

[11] World Health Organization. Integrated management of childhood illness: chart booklet. Geneva, Switzerland: World Health Organization; 2014. Available: https://www.who.int/maternal_child_adolescent/documents/IMCI_chartbooklet/en/. Accessed: 5 May 2025.

[12] Government of Pakistan, World Health Organization, United Nations Children’s Fund. Integrated management of childhood illness: chart booklet. Islamabad, Pakistan: Government of Pakistan; 2019. Available: https://sites.pitt.edu/~super1/Pirzado/Chartbooklet%202019%20Pakistan.pdf. Accessed: 5 May 2025.

[13] World Health Organization. Sexual, reproductive, maternal, newborn, child and adolescent health policy survey 2018–2019: report. Geneva, Switzerland: World Health Organization; 2020. Available: https://iris.who.int/handle/10665/331891. Accessed: 5 May 2025.

[14] Mulholland K Problems with the WHO guidelines for management of childhood pneumonia. Lancet Glob Health. 2018;6:e8–9. 10.1016/S2214-109X(17)30468-0 29241619

[15] World Health Organization. Exploratory meeting to review new evidence for integrated management of childhood illness danger signs, Geneva, Switzerland, 4–5 September 2018. Geneva, Switzerland: World Health Organization; 2019. Available: https://apps.who.int/iris/handle/10665/326100. Accessed: 5 May 2025.

[16] The DHS Program. Service provision assessment (SPA) survey overview. Rockville, MD, USA: ICF International; 2014. Available: https://dhsprogram.com/What-We-Do/Survey-Types/SPA.cfm. Accessed: 12 June 2025.

[17] World Health Organization. Service availability and readiness assessment (SARA): reference manual. Geneva, Switzerland: World Health Organization; 2015. Available: https://www.who.int/data/data-collection-tools/sara. Accessed: 12 June 2025.

[18] Nichols EK Byass P Chandramohan D Clark SJ Flaxman AD Jakob R WHO Verbal Autopsy Working Group The WHO 2016 verbal autopsy instrument: an international standard suitable for automated analysis by InterVA, InSilicoVA, and Tariff 2.0. PLoS Med. 2018;15:e1002486. 10.1371/journal.pmed.1002486 29320495 PMC5761828

[19] Cipam Study Group CIPM Understanding the outcome and management of children aged 2–59 months with chest indrawing pneumonia: a study protocol for an observational study in Ethiopia, India, Nigeria, Pakistan, Uganda and Zambia. BMJ Open. 2024;14:e084350. 10.1136/bmjopen-2024-084350 38904143 PMC11191814

[20] World Health Organization. WHO child growth standards. Geneva, Switzerland: World Health Organization; 2006. Available: https://www.who.int/publications/i/item/924154693X. Accessed: 17 May 2025.

[21] Suhag ZH Pal A Naeem M Ahmed I Khuwaja NA Khakwani S Outcome and management of children with chest indrawing pneumonia at primary health care settings in Pakistan: an observational cohort study. J Glob Health. 2025;15:04096. 10.7189/jogh.15.04096 40153316 PMC11952181

[22] Tigabu Z Toni AT Guadu T Yilma TM Awoke T Engdaw GT Management and outcomes of chest-indrawing pneumonia among children aged 2-59 months in a programme setting in Ethiopia: a prospective observational study. J Glob Health. 2025;15:04217. 10.7189/jogh.15.04217 40755020 PMC12319351

[23] Jacobs C Nkwemu C Ngambi BB Silavwe V Qazi SA Nisar YB Outcomes of children aged 2–59 months with chest indrawing pneumonia managed on an outpatient basis in selected primary health facilities in Zambia. J Glob Health. 2025;15:04089. 10.7189/jogh.15.04089 40643166 PMC12247661

[24] Morre R Sobi K Pameh W Ripa P Vince JD Duke T Safety, effectiveness and feasibility of outpatient management of children with pneumonia with chest indrawing at Port Moresby General Hospital, Papua New Guinea. J Trop Pediatr. 2019;65:71–7. 10.1093/tropej/fmy013 29660106 PMC6366396

[25] Addo-Yobo E Anh DD El-Sayed HF Fox LM Fox MP MacLeod W Outpatient treatment of children with severe pneumonia with oral amoxicillin in four countries: the MASS study. Trop Med Int Health. 2011;16:995–1006. 10.1111/j.1365-3156.2011.02787.x 21545381 PMC3154370

[26] Onono M Abdi M Mutai K Asadhi E Nyamai R Okoth P Community case management of lower chest indrawing pneumonia with oral amoxicillin in children in Kenya. Acta Paediatr. 2018;107:44–52. 10.1111/apa.14405 30570795

[27] Bari A Sadruddin S Khan A Khan I Khan A Lehri IA Community case management of severe pneumonia with oral amoxicillin in children aged 2-59 months in Haripur district, Pakistan: a cluster randomised trial. Lancet. 2011;378:1796–803. 10.1016/S0140-6736(11)61140-9 22078721 PMC3685294

[28] Soofi S Ahmed S Fox MP MacLeod WB Thea DM Qazi SA Effectiveness of community case management of severe pneumonia with oral amoxicillin in children aged 2–59 months in Matiari district, rural Pakistan: a cluster-randomised controlled trial. Lancet. 2012;379:729–37. 10.1016/S0140-6736(11)61714-5 22285055

[29] EMPIC Study Group Innovative, enhanced community management of non-hypoxaemic chest indrawing pneumonia in 2-59-month-old children: a cluster-randomised trial in Africa and Asia. BMJ Glob Health. 2022;7:e006405. 10.1136/bmjgh-2021-006405 34987033 PMC8734014

[30] Angwa LM Ouma C Okoth P Nyamai R Kamau NG Mutai K Acceptability, adherence and clinical outcomes of amoxicillin dispersible tablets versus oral suspension in treatment of children aged 2–59 months with pneumonia in Kenya: a cluster randomised controlled trial. Heliyon. 2020;6:e03786. 10.1016/j.heliyon.2020.e03786 32322742 PMC7160563

[31] Moyano Ariza L Ochoa B Shewade HD Edwards JK Trujillo J Cuellar CM Adherence to guidelines on the use of amoxicillin for treatment of ambulatory pneumonia in children younger than 5 years, Colombia, 2017–2019. Rev Panam Salud Publica. 2023;47:e52. 10.26633/RPSP.2023.52 37082539 PMC10105600

[32] Thi TVL Pham EC Dang-Nguyen DT Evaluation of children’s antibiotic use for outpatient pneumonia treatment in Vietnam. Braz J Infect Dis. 2024;28:103839. 10.1016/j.bjid.2024.103839 38996808 PMC11321292

[33] World Health Organization. The WHO AWaRe (Access, Watch, Reserve) antibiotic book: Practical advice for antibiotic use in common bacterial infections. Geneva, Switzerland: World Health Organization; 2022. Available: https://www.who.int/publications/i/item/9789240062382. Accessed: 5 May 2025.

[34] World Health Organization. Global antimicrobial resistance surveillance system (GLASS) report: early implementation 2017–2018. Geneva, Switzerland: World Health Organization; 2018. Available: https://iris.who.int/bitstream/handle/10665/277175/WHO-WSI-AMR-2018.4-eng.pdf. Accessed: 13 March 2025.

[35] Sulis G Rattanavipapong W Morros-Pita D Ahmad R Cars O Antibiotic use targets in national action plans: sustainability and overlooked resistance drivers. Bull World Health Organ. 2022;100:679–89.

[36] Hsia Y Lee BR Versporten A Yang Y Bielicki J Jackson C Use of the WHO Access, Watch, and Reserve classification to define patterns of hospital antibiotic use (AWaRe): an analysis of paediatric survey data from 56 countries. Lancet Glob Health. 2019;7:e861–71. 10.1016/S2214-109X(19)30071-3 31200888

[37] Hussain H Waters H Omer SB Khan A Baig IY Mistry R The cost of treatment for child pneumonias and meningitis in the Northern Areas of Pakistan. Int J Health Plann Manage. 2006;21:229–38. 10.1002/hpm.847 17044548

[38] Sadruddin S Shehzad S Bari A Khan A Ibad-ul-Haq, Qazi S. Household costs for treatment of severe pneumonia in Pakistan. Am J Trop Med Hyg. 2012;87:137–43. 10.4269/ajtmh.2012.12-0242 23136289 PMC3748514

[39] Hussain H Waters H Khan AJ Omer SB Halsey NA Economic analysis of childhood pneumonia in Northern Pakistan. Health Policy Plan. 2008;23:438–42. 10.1093/heapol/czn033 18755733

[40] Zhang S Incardona B Qazi SA Stenberg K Campbell H Nair H Cost-effectiveness analysis of revised WHO guidelines for management of childhood pneumonia in 74 Countdown countries. J Glob Health. 2017;7:010409. 10.7189/jogh.07.010409 28400955 PMC5344007

[41] Bradley JS Byington CL Shah SS Alverson B Carter ER Harrison C The management of community-acquired pneumonia in infants and children older than 3 months of age: clinical practice guidelines by the Pediatric Infectious Diseases Society and the Infectious Diseases Society of America. Clin Infect Dis. 2011;53:e25–76. 10.1093/cid/cir531 21880587 PMC7107838

[42] National Institute for Health and Care Excellence. Pneumonia in adults and children: diagnosis and management. NICE guideline [NG138]. London, UK: National Institute for Health and Care Excellence; 2019. Available: https://www.nice.org.uk/guidance/ng138. Accessed: 22 July 2025.

[43] Kerai S Nisar I Muhammad I Qaisar S Feroz K Raza A A community-based survey on healthcare utilisation for pneumonia in children in peri-urban slums of Karachi, Pakistan. Am J Trop Med Hyg. 2019;101:1034–41. 10.4269/ajtmh.18-0656 31482784 PMC6838581

[44] Allegranzi B Bagheri Nejad S Combescure C Graafmans W Attar H Donaldson L Burden of endemic health-care-associated infection in developing countries: systematic review and meta-analysis. Lancet. 2011;377:228–41. 10.1016/S0140-6736(10)61458-4 21146207

[45] Rosenthal VD Al-Abdely HM El-Kholy AA Alkhawaja SAA Leblebicioglu H Mehta Y International Nosocomial Infection Control Consortium report, data summary of 50 countries for 2010–2015: device-associated module. Am J Infect Control. 2016;44:1495–504. 10.1016/j.ajic.2016.08.007 27742143

[46] Antimicrobial Resistance Collaborators Global burden of bacterial antimicrobial resistance in 2019: a systematic analysis. Lancet. 2022;399:629–55. 10.1016/S0140-6736(21)02724-0 35065702 PMC8841637

[47] Ginsburg AS Mvalo T Nkwopara E McCollum ED Phiri A Schmicker R Amoxicillin for 3 or 5 days for chest-indrawing pneumonia in Malawian children. N Engl J Med. 2020;383:13–23. 10.1056/NEJMoa1912400 32609979 PMC7233470

[48] Agweyu A Gathara D Oliwa J Muinga N Edwards T Allen E Severe Pneumonia Study Group Oral amoxicillin versus benzyl penicillin for severe pneumonia among Kenyan children: a pragmatic randomised controlled non-inferiority trial. Clin Infect Dis. 2015;60:1216–24. 10.1093/cid/ciu1166 25550349 PMC4370168

[49] Olaniran A Briggs J Pradhan A Bogue E Schreiber B Dini HS Stockouts of essential medicines among community health workers (CHWs) in low- and middle-income countries (LMICs): a systematic literature review of the extent, reasons and consequences. Hum Resour Health. 2022;20:58. 10.1186/s12960-022-00755-8 35840965 PMC9287964

[50] Hussain R Radwan M Habib S Availability and storage conditions of essential medicines at primary healthcare facilities in Punjab, Pakistan. J Ayub Med Coll Abbottabad. 2021;33 Suppl 1:S763–8. 35077623

