# Supplementary material for: Outpatient management of children with chest indrawing pneumonia in primary healthcare settings in Punjab, Pakistan: a prospective cohort study
Source: J Glob Health. 2026 Jun 12;16:04139. doi: 10.7189/jogh.16.04139 (PMC13261329; doi:10.7189/jogh.16.04139)
Supplement: Online Supplementary Document [file jogh-16-04139-s001.pdf]

**Supplement to: Suhag Z, Khuwaja N, Pal A, Naeem M, Naqvi A, Qazi S, Nisar Y. Outpatient management of children with chest indrawing pneumonia in primary healthcare settings in Punjab, Pakistan: a prospective cohort study. J Glob Health. 2026;16:04139.**

# ONLINE SUPPLEMENTARY DOCUMENT

## Supplementary Table 1. Stratified outcomes by nutritional status and immunisation status

A. **Nutritional Status - Under-weight (Weight for Age Z-score) WAZ)**

| Nutritional category | N (total) | Recovered n (%) | Same n (%) | Worsened n (%) | Deaths n (%) | CFR (%) |
|---|---|---|---|---|---|---|
| **Normal (> -2 SD** | 211 | 200(94.79%) | 8(3.79%) | 2(0.95%) | 1(0.47%) | 0.47% |
| **Moderate (-2 to -3 SD)** | 62 | 60(96.77%) | 1(1.61%) | 1(1.61%) | 0(00.0%) | 0.00% |
| **Severe (< -3 SD)** | 83 | 76(91.57%) | 1(1.21%) | 5(6.02%) | 1(1.20%) | 1.20% |

**Nutritional Status - Stunting (Height for Age Z-score ) HAZ)**

| Nutritional category | N (total) | Recovered n (%) | Same n (%) | Worsened n (%) | Deaths n (%) | CFR (%) |
|---|---|---|---|---|---|---|
| **Normal (> –2 SD)** | 230 | 216 (93.91%) | 9 (3.91%) | 4 (1.74%) | 1 (0.43%) | 0.43% |
| **Moderate (–2 to –3 SD)** | 54 | 51 (94.44%) | 0 (0.00%) | 2 (3.70%) | 1 (1.85%) | 1.85% |
| **Severe (< –3 SD)** | 72 | 69 (95.83%) | 1 (1.39%) | 2 (2.78%) | 0 (0.00%) | 0.00% |

**Nutritional Status - Wasting (Weight for Height Z-score — WHZ)**

| Nutritional category | N (total) | Recovered n (%) | Same n (%) | Worsened n (%) | Deaths n (%) | CFR (%) |
|---|---|---|---|---|---|---|
| **Normal (> –2 SD)** | 256 | 244 (95.31%) | 8 (3.13%) | 3 (1.17%) | 1 (0.39%) | 0.39% |
| **Moderate (–2 to –3 SD)** | 44 | 41 (93.18%) | 0 (0.00%) | 3 (6.82%) | 0 (0.00%) | 0.00% |
| **Severe (< –3 SD)** | 56 | 51 (91.07%) | 2 (3.57%) | 2 (3.57%) | 1 (1.78%) | 1.78% |

**Nutritional Status (MUAC)**

| Nutritional category | N (total) | Recovered n (%) | Same n (%) | Worsened n (%) | Deaths n (%)* | CFR (%) |
|---|---|---|---|---|---|---|
| **Normal (≥ 12.5 cm)** | 153 | 145 (94.77%) | 5 (3.27%) | 3 (1.96%) | NA | NA |
| **Moderate (11.5–<12.5 cm)** | 4 | 4 (100.00%) | 0 (0.00%) | 0 (0.00%) | NA | NA |
| **Severe (<11.5 cm)** | 9 | 9 (100.00%) | 0 (0.00%) | 0 (0.00%) | NA | NA |

**Both deaths are younger (less than 6 month there is no MUAC measurement)*

B. **Immunisation Status**

| Immunisation status | N (total) | Recovered n (%) | Same n (%) | Worsened n (%) | Deaths n (%) | CFR (%) |
|---|---|---|---|---|---|---|
| **Fully immunised** | 210 | 199 (94.76%) | 7 (3.33%) | 3 (1.43%) | 1 (0.48%) | 0.47% |
| **Partially immunised** | 136 | 128 (94.12%) | 3 (2.21%) | 4 (2.94%) | 1 (0.74%) | 0.73% |
| **Not immunised** | 10 | 9 (90.00%) | 0 (0.00%) | 1 (10.00%) | 0 (0.00%) | 0.00% |

**Supplementary Table 2. Antibiotics Prescribed by the Healthcare Provider (HCP) at the Primary Healthcare Facility (PHC) at the time of Enrollment (N=356)**

| Name of Medicines | Total | RHC Manga Mandi | BHU Ali Raza Abad | BHU Jallo Pind | BHU Attoki Awan | BHU Manawan |
|---|---|---|---|---|---|---|
| | N=356 | n=131 | n=89 | n=54 | n=41 | n=41 |
| Susp. Amoxicillin | 192 (53.9) | 42 (32.1) | 58 (65.2) | 38 (70.4) | 24 (58.5) | 30 (73.2) |
| Susp. Cefixime | 58 (16.3) | 25 (19.1) | 10 (11.2) | 5 ( 9.3) | 7 (17.1) | 11 (26.8) |
| Susp. Amoxicillin-clavulanic acid | 54 (15.2) | 47 (35.9) | 0 ( 0.0) | 0 ( 0.0) | 7 (17.1) | 0 ( 0.0) |
| Susp. Cotrimoxazole | 33 ( 9.3) | 3 ( 2.3) | 18 (20.2) | 9 (16.7) | 3 ( 7.3) | 0 ( 0.0) |
| Other antibiotics* | 19 ( 5.3) | 14 (10.7) | 3 (3.4) | 2 ( 3.7) | 0 ( 0.0) | 0 ( 0.0) |

BHU – basic health unit, RHC – rural health centre, Susp. – Suspension

*Other antibiotics include 15 who received Susp. azithromycin, three who received Susp. doxycycline, and one who received Susp. cefadroxil

**Supplementary Table 3. Antibiotic Changes Following Non-Response to Initial Treatment Prescribed at Enrollment (n=26)**

| Initial treatment at the time of enrolment | Duration of initial prescribed treatment (days) | Duration of initial prescribed treatment administration (days) | Change in the initial prescribed antibiotic during the follow-up period | Use of new antibiotic treatment (days) | Post-enrolment day/hours at the time of a revisit/hospitalisation | Type of Facility |
|---|---|---|---|---|---|---|
| **Hospitalised Children (n=7) *** | | | | | | |
| Susp. Amoxicillin/Clavulanic Acid | 5 | 2 | Inj. Ceftriaxone | 3 | 3 | Public Hospital |
| Susp. Amoxicillin/Clavulanic Acid | 3 | 1 | Inj. Ceftriaxone, Susp. Cefixime | 3 | 1 | Public Hospital |
| Susp. Amoxicillin | 5 | 5 | Inj. Ceftriaxone | 4 | 13 | Public Hospital |
| Susp. Amoxicillin | 3 | 1 | Inj. Ceftriaxone, Susp. Amoxicillin/Clavulanic Acid | 3 | < 24 hours | Private Hospital |
| Susp. Amoxicillin | 5 | 1 | Inj. Amoxicillin/Clavulanic Acid | 3 | 1 | Public Hospital |
| Susp. Amoxicillin | 5 | 5 | Inj. Ceftriaxone, Susp. Amoxicillin/Clavulanic Acid | 3 | 5 | Public Hospital |
| Susp. Amoxicillin | 5 | 4 | Inj. Ceftriaxone, Susp. Cefixime | 3 | 3 | Private Hospital |
| **Injectable Antibiotic used on an outpatient basis (n=5)** † | | | | | | |

| | | | | | | |
|---|---|---|---|---|---|---|
| Susp. Azithromycin | 3 | 3 | Inj. Ceftriaxone, Inj. Ampicillin | 5 | 4 | Private Hospital |
| Susp. Amoxicillin | 3 | 2 | Inj. Ceftriaxone | 1 | 14 | Primary Health Care Centre |
| Susp. Amoxicillin | 5 | 5 | Inj. Ceftriaxone, Susp. Amoxicillin/Clavulanic Acid | 5 | 5 | Public Hospital |
| Susp. Amoxicillin | 5 | 7 | Inj. Ceftriaxone, Susp. Amoxicillin/Clavulanic Acid | 5 | 7 | Public Hospital |
| Susp. Amoxicillin | 5 | 5 | Inj. Ceftriaxone, Susp. Amoxicillin | 5 | 1 | Public Hospital |
| **Oral antibiotic prescribed at a different outpatient Visit (n=13)** ‡ | | | | | | |
| Susp. Amoxicillin/Clavulanic Acid | 3 | 1 | Susp. Cefixime, Susp. Azithromycin | 3 | 1 | Private Hospital |
| Susp. Cefixime | 5 | 3 | Susp. Cefaclor, Susp. Cefixime | 3 | 1 | Private Hospital |
| Susp. Cefixime | 5 | 2 | Susp. Amoxicillin, Susp. Azithromycin | 5 | 2 | Public Hospital |
| Susp. **Amoxicillin** | **3** | **3** | **Cough Syrup** | **3** | **3** | **Private Clinic** |
| Susp. Cefixime | 4 | 4 | Susp. Cefixime, Susp. Azithromycin | 3 | 4 | Private Clinic |
| Susp. Amoxicillin | 5 | 5 | Susp. Azithromycin | 3 | 10 | Private Clinic |
| Susp. Cefixime | 3 | 3 | Susp. Azithromycin | 3 | 5 | Private Hospital |
| Susp. **Amoxicillin** | **2** | **2** | **Susp. Cefixime** | **5** | **< 24 hours** | **Private Clinic** |
| Susp. Cefixime | 5 | 7 | Susp. Cefixime | 3 | 13 | Private Hospital |
| Susp. Cefixime | 5 | 5 | Susp. Cefixime | 3 | 8 | Private Clinic |
| Susp. Amoxicillin | 5 | 5 | Susp. Amoxicillin | 3 | 6 | Private Clinic |
| Susp. Cefixime | 5 | 5 | Susp. Cefixime | 3 | 6 | Public Hospital |
| Susp. Amoxicillin | 5 | 7 | Susp. Cefixime | 3 | 11 | Private Clinic |

| Oral antibiotic prescribed at one of the study Primary Healthcare Facilities, revisit (n=1) | | | | | | |
|---|---|---|---|---|---|---|
| Susp. Cefixime | 3 | 2 | Susp. Amoxicillin/Clavulanic Acid | 3 | 3 | Primary Health Care Centre |

Inj. – Injection, Susp. – Suspension

* Children who required inpatient care for more than one day during the follow-up period and received at least one dose of injectable antibiotics. Among the seven children with completed follow-up data who were hospitalised during the 15 days post-enrollment, four were brought to a hospital by caregivers, the primary health facility referred two, and a community health worker referred one. Additionally, verbal autopsies for two deceased children confirmed that both were admitted to a hospital before death; however, due to the absence of follow-up forms, they are excluded from the "Hospitalised" breakdown.

† Children who received an Injectable antibiotic on an outpatient basis

‡Children who received other oral antibiotic treatment from another health facility.
